# A nationwide study of aspirin, other non-steroidal anti-inflammatory drugs, and Hodgkin lymphoma risk in Denmark

**DOI:** 10.1038/bjc.2011.443

**Published:** 2011-10-25

**Authors:** E T Chang, T Frøslev, H T Sørensen, L Pedersen

**Affiliations:** 1 Cancer Prevention Institute of California, 2201 Walnut Avenue, Suite 300, Fremont, CA 94538, USA; 2 Division of Epidemiology, Department of Health Research and Policy, Stanford University School of Medicine, HRP Redwood Building, Stanford, CA 94305, USA; 3 Department of Clinical Epidemiology, Aarhus University Hospital, Olof Palmes Allé 43-45, Århus N DK-8200, Denmark

**Keywords:** aspirin, non-steroidal anti-inflammatory drugs, Hodgkin lymphoma, epidemiology, risk, prevention

## Abstract

**Background:**

We recently found an inverse association between low-dose aspirin use and risk of Hodgkin lymphoma (HL) in northern Denmark. To strengthen the evidence for this association, we expanded the study base to include all of Denmark.

**Methods:**

Between 1997 and 2009, 1659 incident HL cases were identified in nationwide databases and matched with ⩽5 population controls on age, sex, and residence. Use of aspirin, selective cyclooxygenase-2 (sCOX-2) inhibitors, and other non-steroidal anti-inflammatory drugs (NSAIDs) from 1995 through 2008 (⩾1 year before the index date) was ascertained via the Danish National Prescription Database. Odds ratios (ORs) for associations with HL risk were estimated using conditional logistic regression.

**Results:**

Ever use (>2 prescriptions) *vs* never/rare use (⩽2 prescriptions) of low-dose aspirin was not associated with HL risk, but the association with long-term use for ⩾7 years *vs* never/rare use was clearly inverse, although statistically nonsignificantly so (OR=0.65, 95% confidence interval (CI): 0.39–1.09). By contrast, ever use of sCOX-2 inhibitors or other NSAIDs (OR=1.27, 95% CI: 1.10–1.47), especially short-term and low- or medium-intensity use, was associated with elevated HL risk.

**Conclusion:**

Our results are consistent with the hypothesis that long-term use of low-dose aspirin, but not other NSAIDs, protects against HL development.

Hodgkin lymphoma (HL) is among the five most common cancers in young adults in western countries (Curado *et al*, 2007). Substantial treatment-related risks of cardiac disease, infertility, and secondary malignancies later in life (Connors, 2005) make primary prevention of HL an important public health goal, yet few, if any, modifiable risk factors for HL are known. Use of aspirin, a non-steroidal anti-inflammatory drug (NSAID), has been hypothesised to protect against HL (Chang *et al*, 2004) by inhibiting the pleiotropic transcription factor nuclear factor kappa-B (NF-*κ*B) (Kopp and Ghosh, 1994), which is constitutively activated in malignant HL cells and required for their survival (Bargou *et al*, 1996). Aspirin and other NSAIDs, as well as selective cyclooxygenase-2 (sCOX-2) inhibitors, may also prevent HL development by suppressing the proliferative, angiogenic effects of COX-2, which is overexpressed in HL (Ohsawa *et al*, 2006).

Using prospectively collected, registry-derived data from ∼1.7 million residents of northern Denmark (Chang *et al*, 2010), we recently confirmed an earlier finding of an inverse association between routine aspirin use and HL risk detected in a retrospective, population-based case–control study in the United States with self-reported past medication use (odds ratio (OR) for use of regular-strength aspirin ⩾2 *vs* <2 times per week=0.60, 95% confidence interval (CI)=0.42–0.85) (Chang *et al*, 2004). However, with a total of 478 HL cases, the results of our study in Denmark had limited statistical precision, and the apparent inverse association was not statistically significant (OR=0.7, 95% CI=0.5–1.2). Therefore, to strengthen our findings by expanding the study base and maximising the sample size, we conducted a nationwide study with prospectively collected, registry-based data to evaluate whether use of aspirin or other NSAIDs may reduce the risk of HL, and combined these results with those from the US study in a meta-analysis.

## Materials and methods

### Study population

This case–control study was nested within the source population of all residents of Denmark between 1 January 1995 and 31 December 2009. During this 14-year period, 6 019 549 unique individuals contributed a total of 75 886 045 person-years. To enable accurate calculation of time at risk, eligible cases and controls were required to be residents of Denmark from 1 January 1995 through the index date (i.e., the date of diagnosis of the case in each matched case–control set). The Danish National Health Service provides tax-supported health care to all residents of Denmark and refunds a portion of patient expenditures on prescription drugs, including aspirin, sCOX-2 inhibitors, and other NSAIDs. All health-related services are registered to patients via their unique central personal registration (CPR) number, which enables linkage between national population-based registries including the Danish Cancer Registry (Storm *et al*, 1997), the Danish National Registry of Patients (Andersen *et al*, 1999), and the Danish National Prescription Database (Gaist *et al*, 1997).

Eligible cases were all individuals diagnosed with first, primary classical HL (excluding nodular lymphocyte predominant HL, which has a distinct aetiology; Appendix 1) between 1 January 1997 and 31 December 2009. We used the Cancer Registry, which includes the CPR number and detailed information for all cancer diagnoses in Denmark since 1943, to identify eligible cases through 2008 (*N*=1494). We used the National Registry of Patients, which includes the CPR number and detailed individual data for all non-psychiatric hospital admissions since 1977 and outpatient contacts since 1995, to ascertain eligible cases diagnosed in 2009 (*N*=165; total cases=1659). The Cancer Registry has secondary histological confirmation of cancer diagnoses from the National Pathology Registry (Sørensen *et al*, 2008; Danish National Board of Health, 2010), whereas the National Registry of Patients contains more recent data.

Within the Danish Civil Registration System database (Pedersen *et al*, 2006), we performed risk-set sampling to select up to five population controls per case among living individuals without a history of HL on the index date. In total, 8089 controls were matched to cases by age, sex, and residence in Denmark on the index date. This study was approved by the Danish Data Protection Board.

### Exposure assessment

The National Prescription Database was established in 1994 and records each customer's CPR number and data on redeemed prescriptions, including prescription type (according to the Anatomical Therapeutic Chemical Classification System (World Health Organization (WHO), 2001); Appendix 1), quantity prescribed, and date of dispensing. The length of each prescription is not recorded; therefore, the dose (e.g., pills prescribed per day) cannot be precisely calculated. By initiating study follow-up in 1995, we ensured at least 2 years of prescription history before the index date of each risk set. We identified prescriptions for low-dose aspirin (75, 100, or 150 mg per tablet), high-dose aspirin (500 mg per tablet), sCOX-2 inhibitors, and other NSAIDs. Prescriptions within 1 year of the index date were excluded from the analysis to reduce bias due to treatment of prediagnostic symptoms or increased surveillance of NSAID users.

Ever users of a medication were defined as those who filled >2 prescriptions for a given medication, and never/rare users were those who filled ⩽2 prescriptions. The average length of a prescription was assumed to be 30 days, based on evidence from studies of gastrointestinal bleeding after use of NSAIDs (e.g., Mellemkjaer *et al*, 2002). Ever users were further classified as recent users or former users, based on whether they had filled >2 or ⩽2 prescriptions during the period 1–2 years before the index date (defined as the ‘recent’ period). Ever users were also defined as long-term or short-term users, based on whether ⩾7 or <7 years elapsed between the first and last prescriptions plus the duration of the last prescription. In addition, ever users were defined as low-intensity (<25%), medium-intensity (25–<50%), or high-intensity (⩾50%) users, based on the estimated total number of days of prescription coverage (using the assumption of 30 days per prescription, on average) divided by the duration of use in days (Robertson *et al*, 2007).

To obtain a nonspecific proxy for chronic use of aspirin, sCOX-2 inhibitors, or other NSAIDs, we used the National Registry of Patients to identify all participants’ inpatient and outpatient diagnoses before the index date, and summarised these using Deyo's adaptation of the Charlson comorbidity index, excluding the categories ‘any tumour,’ ‘metastatic solid tumour,’ and ‘connective tissue disorders’ (Charlson *et al*, 1987; Deyo *et al*, 1992) (Appendix 1). We also used the National Registry of Patients to collect data on history of connective tissue disorders such as rheumatoid arthritis, which is associated with chronic NSAID use and increased HL risk (Blomqvist *et al*, 2000; Ekström *et al*, 2003), before the index date.

### Statistical analysis

We used conditional logistic regression to compute ORs (as estimates of incidence rate ratios) and 95% CIs, matched on age, sex, and residence in Denmark, and additionally adjusted for Charlson comorbidity index (0, 1–2, or ⩾3 comorbidities) and history of connective tissue disorders (yes or no). Further adjustment for region of residence in Denmark did not affect the results (data not shown). Tests for a dose-response trend with increasing intensity of medication use were performed with intensity coded ordinally using the median value within each category. Never/rare users comprised the reference group for all analyses, which were performed with SAS V9.2 (SAS Institute Inc., Cary, NC, USA).

We performed a meta-analysis of results from the US case–control study described above (575 cases, 679 controls (Chang *et al*, 2004)) and results from the present analysis (1659 cases, 8089 controls). Results from the northern Denmark study (Chang *et al*, 2010) were not included due to some overlap with the present study population. Regular *vs* non-regular use of aspirin in the US study was defined as use of regular-strength aspirin (325 mg per tablet) ⩾2 *vs* <2 times per week during the previous 5 years. Thus, the dose and duration of use were higher than those of the variable assessing ever *vs* never/rare low-dose aspirin use in the present study. We analysed the combined exposure variable as either use *vs* non-use or long-term use (5 years in the US study, ⩾7 years in the present study) *vs* non-use of typical-strength aspirin for each study population. Due to differences in the definition of aspirin use between the two studies, we favored a random-effects model of the combined effect, although we also computed fixed-effects models and tested for heterogeneity of effect sizes between the studies. The meta-analysis was performed using Episheet (Rothman, 2008).

## Results

As shown in Table 1, HL cases and their matched controls represented all age groups and geographic regions in Denmark, and were evenly distributed over time. Cases had a higher prevalence of comorbidities and connective tissue disorders than controls.

### Associations with aspirin use

Ever use of low-dose aspirin was not associated with HL risk, and the null association was the same for both former and recent use (Table 2). However, long-term use for ⩾7 years was associated with a substantial 35% reduction in HL risk, although the estimate was statistically nonsignificant (OR=0.65, 95% CI: 0.39, 1.09). When we defined long-term low-dose aspirin use as ⩾5 instead of ⩾7 years of prescription duration, the association was attenuated (OR=0.88, 95% CI: 0.61, 1.27). Intensity of low-dose aspirin use also had an apparent, albeit statistically nonsignificant, inverse dose-response relation with HL risk, and the lowest risk of HL, relative to never/rare users, was among long-term, medium-intensity users of low-dose aspirin (OR=0.57, 95% CI: 0.31, 1.06; 0 cases with long-term, high-intensity use). By contrast, high-dose aspirin use was not associated with HL risk, although the number of ever users in the analysis was small and the OR estimate was therefore unstable.

### Associations with non-aspirin NSAIDs use

Associations with use of sCOX-2 inhibitors and with use of other NSAIDs were similar (data not shown), so results are presented for all non-aspirin NSAIDs combined. Ever *vs* never/rare use of non-aspirin NSAIDs was associated with a statistically significantly increased risk of HL (OR=1.27, 95% CI: 1.10, 1.47), with similar elevations in risk for former and recent, short-term and long-term, and low-intensity and medium-intensity (but not high-intensity) use (Table 2). The strongest detected association was with short-term, medium-intensity use of non-aspirin NSAIDs (OR=1.46, 95% CI: 1.04, 2.06), whereas associations with long-term use were generally positive but statistically nonsignificant.

### Adjusted or stratified associations with aspirin or non-aspirin NSAIDs use

After adjustment for duration and intensity of non-aspirin NSAIDs use, the association with long-term low-dose aspirin use was not substantially altered (OR=0.62, 95% CI: 0.37, 1.04), nor did further adjustment appreciably change the associations with low-dose aspirin use by recentness or intensity of use (data not shown). Conversely, the associations with non-aspirin NSAID use were not meaningfully altered after adjustment for low-dose aspirin use (data not shown). After stratification by use of non-aspirin NSAIDs, we found that among ever users the OR for the association with long-term low-dose aspirin use was 0.46 (95% CI: 0.23, 0.94) (Table 3). Thus, the inverse association between long-term low-dose aspirin use and HL risk was apparently not explained by avoidance of non-aspirin NSAIDs, although this analysis was based on a limited sample size. On the other hand, the positive association with ever use of non-aspirin NSAIDs was detected only among never/rare users of low-dose aspirin (OR=1.23, 95% CI: 1.06, 1.43) and not among ever users (Table 3).

### Secondary stratified associations with aspirin or non-aspirin NSAIDs use

We attempted secondary analyses stratified by age (<40 *vs* ⩾40 years) because the aetiology of HL varies between older and younger adults (MacMahon, 1957). However, the prevalence of low-dose aspirin use in the population under age 40 years was too low to permit statistically stable OR estimates. For non-aspirin NSAIDs, the OR for ever *vs* never/rare use was 0.99 (95% CI: 0.76, 1.30) among those under age 40 years and 1.29 (95% CI: 1.10, 1.52) among those aged 40 years and older. Likewise, positive associations with other categories of non-aspirin NSAID use (including former, short-term, low-intensity, and medium-intensity use) were detected only among adults aged 40 years and older (data not shown).

In secondary analyses restricted to 1223 cases (74%) and 6965 controls (86%) with no history of connective tissue disorder and no Charlson comorbidities, long-term use of low-dose aspirin was no longer associated with HL risk (OR for long-term *vs* never/rare use=1.04, 95% CI: 0.63, 1.74), whereas ever *vs* never/rare use of non-aspirin NSAIDs remained positively associated (OR=1.32, 95% CI: 1.15, 1.51). We also conducted secondary analyses restricted to the 1039 cases (63%) and 5050 controls (62%) with at least 7 years of prescription history and found comparable results (OR for long-term *vs* never/rare use of low-dose aspirin=0.67, 95% CI: 0.40, 1.12; OR for ever *vs* never/rare use of non-aspirin NSAIDs=1.23, 95% CI: 1.04, 1.45). Likewise, after exclusion of prescriptions within 2 years of the index date, the results were equivalent (OR for long-term *vs* never/rare use of low-dose aspirin=0.63, 95% CI: 0.35, 1.14; OR for ever *vs* never/rare use of non-aspirin NSAIDs=1.30, 95% CI: 1.12, 1.52). Finally, in an analysis limited to the 672 cases (41%) diagnosed in the last 5 years of the study period (2005–2009) and their 3270 matched controls (40%), we found similar results (OR for long-term *vs* never/rare use of low-dose aspirin=0.62, 95% CI: 0.35, 1.10; OR for ever *vs* never/rare use of non-aspirin NSAIDs=1.31; 95% CI: 1.08, 1.61).

### Meta-analysis of association with aspirin use

**Figure 1:**
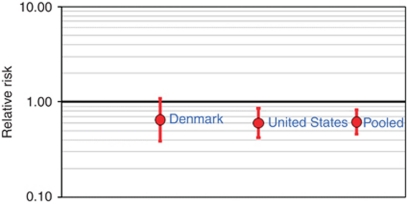
Forest plot for meta-analysis of US and Danish studies of the association between long-term *vs* never/rare aspirin use and risk of Hodgkin lymphoma. The circles show the estimated odds ratios and the vertical lines show the 95% confidence intervals. The summary (pooled) odds ratio was 0.62 (95% confidence interval: 0.46, 0.82) based on both random-effects and fixed-effects models, with no evidence of heterogeneity between studies (*P*=0.80). Study populations included in the meta-analysis were from the United States (565 cases, 679 controls (Chang *et al*, 2004)) and Denmark (1659 cases, 8089 controls; present study). The meta-analysis and forest plot were performed using Episheet (Rothman, 2008).

In a meta-analysis of results from the present study and the previous US case–control study (Chang *et al*, 2004), we found that the summary OR for use *vs* non-use of aspirin was 0.77 (95% CI: 0.49, 1.19) based on a random-effects model (*P* for heterogeneity=0.04; fixed-effects OR=0.82, 95% CI: 0.67, 0.99). If aspirin use in the US study was considered as long-term use, then the summary OR for long-term use *vs* non-use of aspirin was 0.62 (95% CI: 0.46, 0.82), with identical results based on random-effects and fixed-effects models, and no evidence of heterogeneity between the studies (*P*=0.80) (Figure 1).

## Discussion

In this nationwide expansion of our recent nested case–control study in northern Denmark (which covered ∼30% of the present study area) (Chang *et al*, 2010), we substantiated our previous findings by detecting a lower risk of HL among long-term users of low-dose aspirin, compared with never/rare users. This finding is also consistent with earlier findings from the only other analysis, a retrospective case–control study in the United States (Chang *et al*, 2004). Thus, all three existing (to our knowledge) studies of the relation between aspirin use and HL risk have reported at least a statistically nonsignificant inverse association, and a meta-analysis of results from the current and US studies yielded a statistically significant inverse association between long-term aspirin use and HL risk. Taken together, these findings offer considerable evidence worthy of further investigation, especially in large study populations with detailed, prospectively collected information on aspirin use.

Similarly, the observed positive association with use of sCOX-2 inhibitors and other non-aspirin NSAIDs is consistent with our previous finding in northern Denmark. As in our earlier study, the particularly strong association with short-term, medium-intensity use of non-aspirin NSAIDs, as well as the detection of a positive association among aspirin non-users but not among aspirin users, suggests that the apparent effect may be due to use of non-aspirin NSAIDs to treat the fever, night sweats, and swollen lymph nodes that often precede HL diagnosis. Alternatively, the observed association may be due to treatment of another underlying condition that increases HL risk. However, without information on indications for medication use or presence of B symptoms in HL cases, we were unable to explore these possibilities.

Various mechanisms could underlie the observed associations. As mentioned in the introduction, a true protective effect of aspirin against HL may be explained by aspirin-mediated inhibition of constitutively active NF-*κ*B, which leads to spontaneous apoptosis of malignant HL cells (Bargou *et al*, 1996; Izban *et al*, 2001). By contrast, the observed positive association with non-aspirin NSAIDs may be explained by an as-yet unidentified biological pathway. However, we believe that the latter result is more likely explained either by confounding by indication, due to prodromal use of NSAIDs among individuals subsequently diagnosed with HL, or by treatment of underlying inflammatory or infectious medical conditions, or susceptibility to such conditions, that increase the risk of HL. In an attempt to reduce the influence of medical history, we adjusted all analyses for comorbidities and connective tissue disorders, but we remained unable to avoid potential confounding by indication. The lack of an inverse association with low-dose aspirin use among subjects with no Charlson comorbidities or connective tissue disorders may also suggest that certain conditions associated with low-dose aspirin use, rather than aspirin itself, are protective against HL risk. However, cardiovascular disease, for which the vast majority of low-dose aspirin prescriptions in Denmark are ordered (Friis *et al*, 2003), is not known to be inversely associated with HL risk. The association with high-dose aspirin use was unclear, largely due to the low prevalence of use.

Besides the lack of information on indications for prescriptions, our study was limited by our inability to evaluate compliance with prescriptions, as well as the lack of information on over-the-counter use of aspirin and other NSAIDs. However, over-the-counter use of non-aspirin NSAIDs in Denmark represents only 14% of total use (Mellemkjaer *et al*, 2002), and because the Danish government refunds half of the cost of NSAIDs at the time of prescription, it is likely that we captured most physician-recommended use of aspirin and non-aspirin NSAIDs (Friis *et al*, 2003). To the extent that any over-the-counter use would likely be more common among individuals without prescriptions, and perhaps also more common among individuals of high socioeconomic status (which is associated with increased HL risk (Gutensohn and Cole, 1981)), the observed ORs in our study would have underestimated the true associations. Another limitation of our study was the lack of information on prescription length, which prevented us from calculating the precise number of pills prescribed per day or other quantitative measures of medication use. The resultant exposure misclassification probably reduced the statistical precision of our estimates, but was non-differential and, therefore, most likely resulted in underestimated associations.

These limitations are counterbalanced by the many strengths of our study, including the availability of prospectively collected, continuously updated, validated information on drug prescriptions for up to 14 years; the population-based study design with broadly generalisable results; and the complete, validated ascertainment of incident HL. Furthermore, the sample size of 1659 HL cases far exceeds the number of cases included in prior studies of NSAID use and HL risk, and indeed most other epidemiologic studies of HL, thereby contributing to substantial statistical power in our study.

In conclusion, using prospective, population-based data and the largest sample size to date, we found a statistically nonsignificant inverse association between long-term low-dose aspirin use and risk of HL. This finding is consistent with results from two earlier studies, lending further credibility to the hypothesis that aspirin is unique among NSAIDs in protecting against HL. Given that virtually no readily modifiable risk factors for HL have been established, the potential of aspirin to prevent lymphomagenesis is a highly attractive prospect that merits attention in future pooled and other large-scale prospective studies of HL risk.

## Figures and Tables

**Table 1:** Distribution of Hodgkin lymphoma cases and matched controls in Denmark, 1997–2009

	**Cases (*N*=1659), *N* (%)**	**Controls (*N*=8089), *N* (%)**
*Age (years)*		
<20	204 (12)	1030 (13)
20–29	289 (17)	1435 (18)
30–39	296 (18)	1467 (18)
40–49	199 (12)	1005 (12)
50–59	205 (12)	1026 (13)
60–69	212 (13)	1050 (13)
⩾70	254 (15)	1076 (13)
		
*Sex*		
Female	702 (42)	3448 (43)
Male	957 (58)	4641 (57)
		
*Region of residence*		
Nordjylland (North Jutland)	169 (10)	888 (11)
Midtjylland (Central Jutland)	385 (23)	1824 (23)
Syddanmark (Southern Denmark)	368 (22)	1825 (23)
Hovedstaden (Capital Region)	507 (31)	2370 (29)
Sjælland (Zealand)	230 (14)	1182 (15)
		
*Index year*		
1997–2000	467 (28)	2277 (28)
2001–2003	395 (24)	1920 (24)
2004–2006	375 (23)	1827 (23)
2007–2009	422 (25)	2065 (26)
		
*Charlson comorbidity index*		
0	1248 (75)	7029 (87)
1–2	318 (19)	944 (12)
⩾3	93 (6)	116 (1)
		
*Connective tissue disorder*		
No	1599 (96)	7996 (99)
Yes	60 (4)	93 (1)

**Table 2:** Distribution of prescriptions filled by Hodgkin lymphoma cases and matched controls, and ORs with 95% CIs for associations with Hodgkin lymphoma risk in Denmark, 1997–2009

	**Cases (*N*=1659)**	**Controls (*N*=8089)**		
**Prescription medication**	***N* (%)**	***N* (%)**	**OR^a^**	**95% CI**
*Low-dose aspirin*				
Never/rare^b^	1523 (92)	7636 (94)	1.00	(Reference)
Ever^b^	136 (8)	453 (6)	0.94	(0.74–1.19)
				
Former^c^	39 (2)	134 (2)	0.91	(0.61, 1.36)
Recent^c^	97 (6)	319 (4)	0.95	(0.72, 1.24)
				
Short term^d^	114 (7)	362 (4)	1.01	(0.78, 1.31)
Long term^d^	22 (1)	91 (1)	0.65	(0.39, 1.09)
				
Low intensity^e^	38 (2)	114 (1)	1.08	(0.72, 1.63)
Medium intensity^e^	93 (6)	317 (4)	0.90	(0.58, 1.19)
High intensity^e^	5 (0.3)	22 (0.3)	0.77	(0.27, 2.15)
			*P*_trend_=0.20	
Low intensity^e^/short term^d^	30 (2)	92 (1)	1.13	(0.72, 1.77)
Medium intensity^e^/short term^d^	8 (0.5)	22 (0.3)	0.99	(0.74, 1.33)
High intensity^e^/short term^d^	79 (5)	250 (3)	0.83	(0.30, 2.33)
Low intensity^e^/long term^d^	14 (1)	67 (1)	0.92	(0.38, 2.18)
Medium intensity^e^/long term^d^	5 (0.3)	20 (0.2)	0.57	(0.31, 1.06)
High intensity^e^/long term^d^	0 (0)	2 (0.02)	—	
				
*High-dose aspirin*				
Never/rare^b^	1653 (100)	8075 (100)	1.00	(Reference)
Ever^b^	6 (0.4)	14 (0.2)	1.46	(0.54, 3.97)
				
*Selective COX-2 inhibitors or other NSAIDs*				
Never/rare^b^	1263 (76)	6569 (81)	1.00	(Reference)
Ever^b^	396 (24)	1520 (19)	1.27	(1.10, 1.47)
				
Former^c^	306 (18)	1189 (15)	1.27	(1.09, 1.49)
Recent^c^	90 (5)	331 (4)	1.25	(0.96, 1.62)
				
Short term^d^	269 (16)	1021 (13)	1.29	(1.10, 1.51)
Long term^d^	127 (8)	499 (6)	1.21	(0.96, 1.54)
				
Low intensity^e^	280 (17)	1101 (14)	1.28	(1.09, 1.51)
Medium intensity^e^	69 (4)	218 (3)	1.47	(1.09, 1.98)
High intensity^e^	47 (3)	201 (2)	0.99	(0.70, 1.40)
			*P*_trend_=0.58	
Low intensity^e^/short term^d^	176 (11)	682 (8)	1.30	(1.07, 1.57)
Medium intensity^e^/short term^d^	104 (6)	419 (5)	1.46	(1.04, 2.06)
High intensity^e^/short term^d^	49 (3)	163 (2)	1.09	(0.76, 1.55)
Low intensity^e^/long term^d^	20 (1)	55 (1)	1.24	(0.96, 1.60)
Medium intensity^e^/long term^d^	44 (3)	176 (2)	1.45	(0.83, 2.54)
High intensity^e^/long term^d^	3 (0.2)	25 (0.3)	0.39	(0.11, 1.41)

Abbreviations: CI=confidence intervals; COX-2=cyclooxygenase-2; NSAID=non-steroidal anti-inflammatory drug; OR=odds ratio.

a Adjusted for age, sex, Charlson comorbidity index, and history of connective tissue disorder.

b Never/rare use: ⩽2 prescriptions total; ever use: >2 prescriptions total.

c Recent use: >2 prescriptions within the period 1–2 years before index date; former use: >2 prescriptions overall, but ⩽2 during the recent period.

d Short-term use: <7 years between first prescription and end of last prescription; long-term use: ⩾7 years between first prescription and end of last prescription.

e Low-intensity use: <25% prescription coverage during total duration of use; medium-intensity use: 25–<50% prescription coverage; high-intensity use: ⩾50% prescription coverage.

**Table 3:** Distribution of prescriptions filled by Hodgkin lymphoma cases and matched controls, and ORs with 95% CIs for associations with Hodgkin lymphoma risk in Denmark, 1997–2009, stratified by ever *vs* never/rare use of low-dose aspirin or non-aspirin NSAIDs

	**Cases (*N*=1659)**	**Controls (*N*=8089)**		
**Prescription medication**	***N* (%)**	***N* (%)**	**OR^a^**	**95% CI**
*Low-dose aspirin*				
Ever users of selective COX-2 inhibitors or other NSAIDs				
Never/rare^b^	330 (20)	1326 (16)	1.00	(Reference)
Short term^c^	55 (3)	141 (2)	0.95	(0.64, 1.40)
Long term^c^	11 (1)	53 (1)	0.46	(0.23, 0.94)
Never/rare users of selective COX-2 inhibitors or other NSAIDs				
Never/rare^b^	1193 (72)	6310 (78)	1.00	(Reference)
Short term^c^	59 (4)	221 (3)	1.00	(0.72, 1.39)
Long term^c^	11 (1)	38 ()	1.06	(0.53, 2.15)
				
*Selective COX-2 inhibitors or other NSAIDs*				
Ever users of low-dose aspirin				
Never/rare^b^	70 (4)	259 (3)	1.00	(Reference)
Ever^b^	66 (4)	194 (2)	0.97	(0.64, 1.46)
Never users of low-dose aspirin				
Never/rare^b^	1193 (72)	6310 (78)	1.00	(Reference)
Ever^b^	330 (20)	1326 (16)	1.23	(1.06, 1.43)

Abbreviations: CI=confidence intervals; COX-2=cyclooxygenase-2; NSAID=non-steroidal anti-inflammatory drug; OR=odds ratio.

a Adjusted for age, sex, Charlson comorbidity index, and history of connective tissue disorder.

b Never/rare use: ⩽2 prescriptions total; ever use: >2 prescriptions total.

c Short-term use: <7 years between first prescription and end of last prescription; long-term use: ⩾7 years between first prescription and end of last prescription.

**Table A1 tblA1:** 

	**ATC codes**	**ICD-8 codes**	**ICD-10 codes**
*Outcome*			
Hodgkin lymphoma			C81, morphologies M965-M966
			
*Exposure*			
Low-dose aspirin	B01AC06, N02BA01		
High-dose aspirin	N02BA51, N02BA01		
Selective cyclooxygenase-2 inhibitors	M01AH01, M01AH02, M01AH03, M01AH05, M01AC05, M01AB05, M01AC06		
Other non-steroidal anti-inflammatory drugs	All other codes within group M01A		
			
*Comorbidity*			
Myocardial infarction		410	I21; I22; I23
Congestive heart failure		427.09; 427.10; 427.11; 427.19; 428.99; 782.49	I50; I11.0; I13.0; I13.2
Peripheral vascular disease		440; 441; 442; 443; 444; 445	I70; I71; I72; I73; I74; I77
Cerebrovascular disease		430-438	I60-I69; G45; G46
Dementia		290.09-290.19; 293.09	F00-F03; F05.1; G30
Chronic pulmonary disease		490-493; 515-518	J40-J47; J60-J67; J68.4; J70.1; J70.3; J84.1; J92.0; J96.1; J98.2; J98.3
Ulcer disease		530.91; 530.98; 531-534	K22.1; K25-K28
Mild liver disease		571; 573.01; 573.04	B18; K70.0-K70.3; K70.9; K71; K73; K74; K76.0
Diabetes type1		249.00; 249.06; 249.07; 249.09	E10.0, E10.1; E10.9
Diabetes type2		250.00; 250.06; 250.07; 250.09	E11.0; E11.1; E11.9
Hemiplegia		344	G81; G82
Moderate to severe renal disease		403; 404; 580-583; 584; 590.09; 593.19; 753.10-753.19; 792	I12; I13; N00-N05; N07; N11; N14; N17-N19; Q61
Diabetes with end organ damage			
Type1		249.01-249.05; 249.08	E10.2-E10.8
Type2		250.01-250.05; 250.08	E11.2-E11.8
			
Leukemia		204-207	C91-C95
Lymphoma		200-203; 275.59	C81-C85; C88; C90; C96
Moderate to severe liver disease		070.00; 070.02; 070.04; 070.06; 070.08; 573.00; 456.00-456.09	B15.0; B16.0; B16.2; B19.0; K70.4; K72; K76.6; I85
AIDS		079.83	B21-B24

Abbreviations: ATC=anatomical therapeutic chemical; ICD=International Classification of Diseases.

## Appendix 1

### Diagnosis and medication codes

[Table tblA1]

## References

[bib1] Andersen TF, Madsen M, Jorgensen J, Mellemkjoer L, Olsen JH (1999) The Danish National Hospital register. A valuable source of data for modern health sciences. Dan Med Bull 46: 263–268 10421985

[bib2] Bargou RC, Leng C, Krappmann D, Emmerich F, Mapara MY, Bommert K, Royer HD, Scheidereit C, Dorken B (1996) High-level nuclear NF-kappa B and Oct-2 is a common feature of cultured Hodgkin/Reed-Sternberg cells. Blood 87: 4340–4347 8639794

[bib3] Blomqvist P, Feltelius N, Ekbom A, Klareskog L (2000) Rheumatoid arthritis in Sweden. Drug prescriptions, costs, and adverse drug reactions. J Rheumatol 27: 1171–1177 10813283

[bib4] Chang ET, Cronin-Fenton DP, Friis S, Hjalgrim H, Sørensen HT, Pedersen L (2010) Aspirin and other nonsteroidal anti-inflammatory drugs in relation to Hodgkin lymphoma risk in northern Denmark. Cancer Epidemiol Biomarkers Prev 19: 59–64 20056623 10.1158/1055-9965.EPI-09-0909 PMC2837543

[bib5] Chang ET, Zheng T, Weir EG, Borowitz M, Mann RB, Spiegelman D, Mueller NE (2004) Aspirin and the risk of Hodgkin's lymphoma in a population-based case-control study. J Natl Cancer Inst 96: 305–315 14970279 10.1093/jnci/djh038

[bib6] Charlson ME, Pompei P, Ales KL, MacKenzie CR (1987) A new method of classifying prognostic comorbidity in longitudinal studies: development and validation. J Chronic Dis 40: 373–383 3558716 10.1016/0021-9681(87)90171-8

[bib7] Connors JM (2005) State-of-the-art therapeutics: Hodgkin's lymphoma. J Clin Oncol 23: 6400–6408 16155026 10.1200/JCO.2005.05.016

[bib8] Curado MP, Edwards B, Shin HR, Storm H, Ferlay J, Heanue M, Boyle P (eds). (2007) Cancer Incidence in Five Continents, Vol. IX. IARC Scientific Publications: Lyon. Data available online at: http://ci5.iarc.fr/CI5i-ix/ci5i-ix.htm

[bib9] Danish National Board of Health (2010) [The Cancer Registry 2009]. Danish National Board of Health: Copenhagen. Available at: http://www.sst.dk/publ/Publ2010/DOKU/Registre/Cancerregisteret2009.pdf

[bib10] Deyo RA, Cherkin DC, Ciol MA (1992) Adapting a clinical comorbidity index for use with ICD-9-CM administrative databases. J Clin Epidemiol 45: 613–619 1607900 10.1016/0895-4356(92)90133-8

[bib11] Ekström K, Hjalgrim H, Brandt L, Baecklund E, Klareskog L, Ekbom A, Askling J (2003) Risk of malignant lymphomas in patients with rheumatoid arthritis and in their first-degree relatives. Arthritis Rheum 48: 963–970 12687538 10.1002/art.10939

[bib12] Friis S, Sorensen HT, McLaughlin JK, Johnsen SP, Blot WJ, Olsen JH (2003) A population-based cohort study of the risk of colorectal and other cancers among users of low-dose aspirin. Br J Cancer 88: 684–688 12618874 10.1038/sj.bjc.6600760 PMC2376336

[bib13] Gaist D, Sorensen HT, Hallas J (1997) The Danish prescription registries. Dan Med Bull 44: 445–448 9377907

[bib14] Gutensohn N, Cole P (1981) Childhood social environment and Hodgkin's disease. New Engl J Med 304: 135–140 6255329 10.1056/NEJM198101153040302

[bib15] Izban KF, Ergin M, Huang Q, Qin JZ, Martinez RL, Schnitzer B, Ni H, Nickoloff BJ, Alkan S (2001) Characterization of NF-kappaB expression in Hodgkin's disease: inhibition of constitutively expressed NF-kappaB results in spontaneous caspase-independent apoptosis in Hodgkin and Reed-Sternberg cells. Mod Pathol 14: 297–310 11301346 10.1038/modpathol.3880306

[bib16] Kopp E, Ghosh S (1994) Inhibition of NF-kappa B by sodium salicylate and aspirin. Science 265: 956–959 8052854 10.1126/science.8052854

[bib17] MacMahon B (1957) Epidemiological evidence on the nature of Hodgkin's disease. Cancer 10: 1045–1054 13472655 10.1002/1097-0142(195709/10)10:5<1045::aid-cncr2820100527>3.0.co;2-0

[bib18] Mellemkjaer L, Blot WJ, Sorensen HT, Thomassen L, McLaughlin JK, Nielsen GL, Olsen JH (2002) Upper gastrointestinal bleeding among users of NSAIDs: a population-based cohort study in Denmark. Br J Clin Pharmacol 53: 173–181 11851641 10.1046/j.0306-5251.2001.01220.x PMC1874281

[bib19] Ohsawa M, Fukushima H, Ikura Y, Inoue T, Shirai N, Sugama Y, Suekane T, Kitabayashi C, Nakamae H, Hino M, Ueda M (2006) Expression of cyclooxygenase-2 in Hodgkin's lymphoma: its role in cell proliferation and angiogenesis. Leuk Lymphoma 47: 1863–1871 17064999 10.1080/10428190600685442

[bib20] Pedersen CB, Gotzsche H, Moller JO, Mortensen PB (2006) The Danish Civil Registration system. A cohort of eight million persons. Dan Med Bull 53: 441–449 17150149

[bib21] Robertson DJ, Larsson H, Friis S, Pedersen L, Baron JA, Sorensen HT (2007) Proton pump inhibitor use and risk of colorectal cancer: a population-based, case-control study. Gastroenterology 133: 755–760 17678921 10.1053/j.gastro.2007.06.014

[bib22] Rothman K (2008) Episheet. Spreadsheets for the Analysis of Epidemiologic Data. Version of 11 June 2008. Available at: http://krothman.byethost2.com/Episheet.xls

[bib23] Sørensen HT, Christensen T, Schlosser HK, Pedersen L (eds). (2008) Use of Medical Databases in Clinical Epidemiology. Department of Clinical Epidemiology, Aarhus University Hospital: Aarhus, Denmark

[bib24] Storm HH, Michelsen EV, Clemmensen IH, Pihl J (1997) The Danish Cancer Registry – history, content, quality and use. Dan Med Bull 44: 535–539 9408738

[bib25] World Health Organization (WHO) (2001) ATC/DDD classification. In WHO Drug Information, vol. 15, No. 2, pp 84–88. World Health Organization: Geneva

